# Effects of Different Molecular Weight Polysaccharides From *Dendrobium officinale* Kimura & Migo on Human Colorectal Cancer and Transcriptome Analysis of Differentially Expressed Genes

**DOI:** 10.3389/fphar.2021.704486

**Published:** 2021-12-03

**Authors:** Shengchang Tao, Zhiyao Ren, Zerui Yang, Shuna Duan, Zhongxian Wan, Jiahui Huang, Chenxing Liu, Gang Wei

**Affiliations:** ^1^ School of Pharmaceutical Sciences, Guangzhou University of Chinese Medicine, Guangzhou, China; ^2^ Department of Pharmacy, Affiliated Dongguan Hospital, Southern Medical University, Dongguan, China; ^3^ Department of Systems Biomedical Sciences, School of Medicine, Jinan University, Guangzhou, China; ^4^ NHC Key Laboratory of Male Reproduction and Genetics, Guangzhou, China; ^5^ Department of Central Laboratory, Family Planning Research Institute of Guangdong Province, Guangzhou, China; ^6^ Shaoguan Institute of Danxia Dendrobium Officinale, Shaoguan, China

**Keywords:** *Dendrobium officinale* Kimura & Migo, polysaccharide, molecular weight, colorectal cancer, *Astragalus membranaceus* polysaccharides, *Lentinus edodes* polysaccharides, RNA sequencing

## Abstract

We investigated the antitumor effects of four fractions of *Dendrobium officinale* Kimura & Migo (*D*. *officinale*) polysaccharides with different molecular weights (Mw), *Astragalus membranaceus* polysaccharides (APS) and *Lentinus edodes* polysaccharides (LNT) on colorectal cancer (CRC) using a zebrafish xenograft model. Transcriptome sequencing was performed to further explore the possible antitumor mechanisms of *D*. *officinale* polysaccharides. Fractions of *D*. *officinale* polysaccharides, LNT, and APS could significantly inhibit the growth of HT-29 cells in a zebrafish xenograft model. One fraction of *D*. *officinale* polysaccharides called DOPW-1 (Mw of 389.98 kDa) exhibited the strongest tumor inhibition. Compared with the control group, RNA-seq revealed that the DOPW-1–treated experimental group had 119 differentially expressed genes (DEGs), of which 45 had upregulated expression and 74 had downregulated expression. Analyses using Gene Ontology and Kyoto Encyclopedia of Genes and Genomes suggested that the pathway “apoptosis-multiple species” was the most significantly enriched. Our data indicated that 1) fractions of *D*. *officinale* polysaccharides of Mw 389.98 kDa were most suitable against CRC; 2) DOPW-1 could be developed into a clinical agent against CRC; and 3) an apoptosis pathway is important for DOPW-1 to inhibit the proliferation of HT-29 cells.

## Introduction

*Dendrobium officinale* Kimura & Migo (*D*. *officinale*) is a perennial herb in the Orchidaceae family. It possesses high pharmacological value and nutritional value. *D*. *officinale* has been used in traditional Chinese medicine formulations and a functional food in China for more than 1,000 years (Ng et al., 2012).

*D*. *officinale* polysaccharides possess anticancer (Yu et al., 2018a; Zhang et al., 2019; Zhang et al., 2020; Tao et al., 2021), immunomodulation (Huang et al., 2016; Tao et al., 2019), antioxidant (Luo et al., 2016), hypoglycemic (Wang et al., 2018; Kuang et al., 2020) and antifatigue (Wei et al., 2017) activities. However, few studies have focused on the activity of *D*. *officinale* polysaccharides against colorectal cancer (CRC).

The molecular weight (Mw) of polysaccharides has been considered to have a close relationship with the biological activities of polysaccharides (Wasser, 2002). Wang et al. (2014) showed that high-Mw polysaccharides (524 kDa) from *Lentinus edodes* displayed robust antitumor effects. Ren et al. (2016) revealed that a high-Mw polysaccharide (463 kDa) from *Pleurotus eryngii* had antitumor effects against HepG2 cells. However, Xu et al. (2016) revealed that medium-Mw polysaccharides (rather than high-Mw polysaccharides) from *Camellia*-species seed cake displayed excellent antioxidant activity. Bhadja et al. (2016) revealed that polysaccharides from seaweed with low Mw had a repairing effect on damaged HK-2 cells. Unfortunately, the relationship between the Mw and biological activity of *D*. *officinale* polysaccharides has not been investigated deeply. Zhang et al. (2019) explored the suitable Mw range of polysaccharides from *D*. *officinale* on inducing the apoptosis of HeLa cells, but was studied *in vitro* only. Therefore, for CRC, the Mw of *D*. *officinale* polysaccharides that are most suitable *in vivo* is still unknown.

In China and Japan, lentinan injection (which contains the main active ingredients of *Lentinus edodes* polysaccharides (LNT)) has been approved as adjuvant therapy for malignant tumors (Sun et al., 2020). *Astragalus membranaceus* polysaccharides (APS) have been applied widely in the clinic due to their immunomodulatory and antitumor activities (Zhou et al., 2017). Therefore, comparing the anticancer effects of *D*. *officinale* polysaccharides with those of APS and LNT could help provide a scientific basis for developing the polysaccharides of *D*. *officinale* as pharmacological agents.

Zebrafish (*Danio rerio*) is regarded as an “ideal” model organism for assessing human diseases because it possesses several orthologs with human genes and drug targets (Gunnarsson et al., 2008; Howe et al., 2013; Kim et al., 2013; Dai et al., 2014). Hence, zebrafish could be used to test the effects of *D*. *officinale* polysaccharides.

Transcriptome studies provide a promising perspective for exploring the molecular mechanisms of drug functions and investigating the underlying pathogenesis of diseases and modes of drug action (Wang et al., 2008; Li et al., 2011; Chen et al., 2012). Transcriptome abundance is measured by RNA sequencing (RNA-seq) (Hao et al., 2018). Some transcriptome analyses that aimed at elucidating the molecular mechanisms related to the antitumor effects of polysaccharides have been undertaken. For example, Kang et al. (2016) revealed the underlying antitumor mechanism of marine algae (*Gracilariopsis lemaneiformis*) polysaccharides by transcriptome profiling. Ren et al. (2019) uncovered the underlying antitumor mechanism of the action of polysaccharides from dandelions in inhibiting a specific pathway: phosphoinositide 3-kinase/protein kinase B/mammalian target of rapamycin. Qi et al. (2020) exposed the underlying anti-CRC mechanism of *Cordyceps sinensis* polysaccharides. Wang et al. (2019) revealed the underlying antitumor mechanism of the polysaccharides of Taishan *Pinus massoniana* pollen. All these research teams used RNA-seq to provide these observations.

We compared the effects of *D*. *officinale* polysaccharides of different Mw, as well as LNT and APS, on the growth of HT-29 cells in a zebrafish xenograft model. Also, we evaluated the changes in the gene-expression profile induced by treatment with *D*. *officinale* polysaccharides on HT-29 cells by RNA-seq to explore the underlying anti-CRC mechanism.

## Materials and Methods

### Ethical Approval of the Study Protocol

Animal care and experimental protocols were approved (IACUC-2019-194, IACUC-2020-097) by the Animal Ethics Committee at Hunter Biotechnology (Hangzhou, China) and were conducted in accordance with the Chinese Association for Laboratory Animal Sciences guidelines.

### Materials

5-Fluorouracil (5-Fu) was purchased from Sigma-Aldrich (Saint Louis, MO, United States). APS was obtained from Tianjin Cinorch Pharmaceuticals (Tianjin, China). LNT was purchased from Jinling Pharmaceuticals (Jinling, China). Also, 5× All-In-One RT MasterMix (with AccuRT Genomic DNA Removal Kit) and EvaGreen 2× quantitative polymerase chain reaction (qPCR) MasterMix-LOW ROX were purchased from Applied Biological Materials (Vancouver, Canada). The TRIzol^®^ reagent was obtained from CoWin Biosciences (Beijing, China).

### Cell Lines

A human CRC cell line (HT-29) was purchased from the Cell Line Bank of the Chinese Academy of Sciences (Shanghai, China). HT-29 cells were cultured in McCoy’s 5A medium (Gibco, Grand Island, NY, United States) containing 10% fetal bovine serum (Gibco), penicillin (100 U·ml^−1^), and streptomycin (100 μg·ml^−1^) at 37°C in a humidified atmosphere with 5% CO_2_.

### Zebrafish

Wild-type AB zebrafish maintained at Hunter Biotechnology was incubated at 28°C with E3 medium (pH 7.2) under a 14 h: 10 h light-dark cycle according to a standard zebrafish breeding protocol (Kimmel et al., 1995). The embryos were obtained by natural spawning.

### Polysaccharides From *Dendrobium officinale* Kimura & Migo

Three polysaccharide fractions, DOP2 (D2), DOP8 (D8), and DOP122 (D122), with a Mw (in kDa) of 24.89, 80.32, and 1,224.54, respectively, were obtained from the stems of *D*. *officinale*, as described previously (Zhang et al., 2020). In brief, after hot water extraction, a *D*. *officinale*–extracting solution was precipitated with ethanol followed by oxidative degradation with a Vc–H_2_O_2_–FeCl_2_·4H_2_O system. Then, D2, D8, and D122 were obtained by freeze-drying.

Another polysaccharide, DOPW-1, of Mw 389.98 kDa, was isolated from the stems of *D*. *officinale* according to a method described previously (Tao et al., 2019). In brief, after hot water extraction, a *D*. *officinale*–extracting solution was precipitated with ethanol followed by purification using a DEAE cellulose-52 column and Sephadex G-300 HR column. Then, DOPW-1 was obtained by freeze-drying.

### Effects of Polysaccharides of Different Molecular Weight in a Zebrafish Xenograft Model

A zebrafish xenograft model was established according to the method described by Hamilton et al. (2016). In brief, zebrafish xenografts were generated by microinjection of ∼200 chloromethylbenz-amino derivatives of 1,1′-dioctadecyl-3,3,3′,3′-tetramethylindocarbocyanine perchlorate (CM-Dil)–labeled HT-29 cells into the yolk sac of zebrafish embryos 2 days post fertilization (dpf).

After establishing the zebrafish xenograft model, the embryos (3 dpf) were placed into six groups randomly: model; 5-Fu (50 ng/zebrafish); D2 (250 μg·ml^−1^); D8 (250 μg·ml^−1^); DOPW-1 (250 μg·ml^−1^); D122 (250 μg·ml^−1^). The embryos were transferred into six-well plates (30 embryos/well). The dose of 5-Fu was selected based on the study by Xu et al. (2019), and the dose of experimental groups was selected based on our previous study (Tao et al., 2021), where we explored the maximum tolerance concentration (MTC) of polysaccharides from *D*. *officinale* and determined the effect of polysaccharides from *D*. *officinale* (27.8, 83.3, 250 μg·ml^−1^) in the zebrafish xenograft model. The results indicated that the concentration of 250 μg·ml^−1^ is determined as MTC and it has the most significant inhibitory effect on CRC. Subsequently, 5-Fu was administered through caudal vein microinjection. D2, D8, DOPW-1, and D122 were dissolved in the embryo medium. 2 days after administration, fluorescence images were captured using a fluorescence microscope (AZ100; Nikon, Japan). The fluorescence intensity was quantified with NIS-Elements D 3.10 (Nikon). The inhibition of tumor growth was calculated according to the following formula:

$$\text{Inhibition of tumor growth }\left(\text{\%}\right)=\left(1-\frac{\text{fluorescence intensity of experimental group}}{\text{fluorescence intensity of model group}}\right)\times 100\%.$$

### Effects of *Astragalus membranaceus* Polysaccharides, *Dendrobium officinale* Kimura & Migo Polysaccharide-1, and *Lentinus edodes* Polysaccharides in a Zebrafish Xenograft Model

After establishing the zebrafish xenograft model, the embryos (3 dpf) were divided randomly into five groups: model; 5-Fu (50 ng/zebrafish); APS (250 μg·ml^−1^); DOPW-1 (250 μg·ml^−1^); LNT (250 μg·ml^−1^). The embryos were transferred into six-well plates (30 embryos/well). Subsequently, 5-Fu was administered through caudal vein microinjection. APS, DOPW-1, and LNT were dissolved in the embryo medium. 2 days after administration, and fluorescence images were captured using a fluorescence microscope (AZ100). The fluorescence intensity was quantified with NIS-Elements D 3.10 (Nikon). Inhibition of tumor growth was calculated as described in the previous section.

### RNA Extraction, Library Preparation, and Transcriptome Sequencing

In our previous study, cell proliferation assay was conducted (Tao et al., 2021), and the results demonstrated that the concentration of 400 μg·ml^−1^ possess the most significant inhibitory effect on HT-29 cells. Therefore, 400 μg·ml^−1^ was selected as the intervention dosage in this experiment. In brief, HT-29 cells were seeded in six-well plates (1×10^6^ cells/well) for 24 h, and treated/ untreated with DOPW-1 (400 μg·ml^−1^) for an additional 48 h. After cell harvesting, total RNA was extracted using the TRIzol reagent at 4°C, and the concentration and purity (optical density at 260 nm (OD_260_)/OD_280_ ≥ 1.8, OD_260_/OD_230_ ≥ 1.5) were tested using a spectrophotometer (NanoDrop™ 2000; Thermo Fisher Scientific, Wilmington, DE, United States). The RNA Nano 6000 Assay Kit of the Bioanalyzer 2,100 system (Agilent Technologies, Santa Clara, CA, United States) was used to evaluate RNA integrity to ensure that the RNA integrity number ≥8.0. Finally, extracted RNA samples were frozen immediately at −80°C until use.

According to the manufacturer’s recommendations, the NEBNext® Ultra™ RNA Library Prep Kit for Illumina® (New England Biolabs, Ipswich, MA, United States) was used to construct the sequencing libraries. This was achieved using 1 μg of total RNA from each sample, and then index codes were added to attribute sequences to each sample. Clustering of the index-coded samples was performed using the TruSeq PE Cluster Kit v4-cBot-HS (Illumina, San Diego, CA, United States) on a cBot Cluster Generation System in accordance with the manufacturer’s (Illumina) directions. After cluster generation, sequencing of libraries was performed on an Illumina platform, and paired-end reads were generated (Chao et al., 2019).

### Bioinformatics Analysis

Raw data with the “fastq” format were analyzed using in-house Perl scripts. In this step, the adapter sequence, poly-N-containing reads, and low-quality reads in raw data were discarded to obtain clean data. Meanwhile, Q20, Q30, GC-content, and sequence duplication in clean data were measured for quality control. Then, the filtered clean data of high quality were used for further analyses.

After the raw sequences had been processed and converted to clean reads, the latter were mapped to the reference genome sequence using HISAT2. Only reads with perfect matches or single mismatches were utilized further for this analysis based on the reference genome.

Several databases were used to annotate gene function: Nr (National Center for Biotechnology Information non-redundant protein sequences, ftp://ftp.ncbi.nih.gov/blast/db/); Pfam (Protein Family, http://pfam.xfam.org/); COG (Clusters of Orthologous Groups of Proteins, www.ncbi.nlm.nih.gov/COG/); KOG (Clusters of Protein Homology, www.ncbi.nlm.nih.gov/KOG/); SWISS-PROT (manually annotated and reviewed protein sequences, http://www.expasy.ch/sprot); KO (Kyoto Encyclopedia of Genes and Genomes (KEGG) Ortholog, http://www.genome.jp/kegg/); GO (Gene Ontology, www.geneontology.org/).

Fragments per kilobase of transcript per million mapped (FPKM) values were used to estimate gene expression. FPKM was calculated using the following formula:

$$\text{FPKM}=\frac{\text{cDNA Fragments}}{\text{Mapped Fragments}\left(\text{Millions}\right)\text{*TranscriptLength}\left(\text{kb}\right)}.$$

Analysis of differential expression of the two groups was executed using edgeR (Robinson et al., 2010). Differentially expressed genes (DEGs) with *P* < 0.05 and fold change ≥2 were screened for further analysis. Analysis of the functional enrichment of DEGs using the GO database was performed using GOseq within R (R Institute for Statistical Computing, Vienna, Austria) based on Wallenius’ noncentral hyper-geometric distribution (Young et al., 2010), which can adjust for bias in gene length in DEGs. KOBAS (Mao et al., 2005) was applied for the analysis of pathway enrichment of DEGs using the KEGG database.

### Analysis of Gene Expression by Real-Time Reverse Transcription-Quantitative Polymerase Chain Reaction

HT-29 cells were seeded in six-well plates at 1×10^6^ cells/well for 24 h, and then treated or not treated with DOPW-1 (400 μg·ml^−1^) for an additional 48 h. After cell harvesting, total RNA was extracted using the TRIzol reagent. The quantity and quality of cells were evaluated using a spectrophotometer (NanoDrop 2000). RNA was transcribed into complementary DNA using the 5× All-In-One RT MasterMix (with AccuRT Genomic DNA Removal Kit) according to the manufacturer’s instructions. Expression of apoptosis-related genes (Bax, B-cell lymphoma (Bcl)-2, P53, caspase-3, cytochrome C) was detected using a real-time PCR instrument (ABI 7500; Applied Biosystems, Foster City, CA, United States) with EvaGreen 2× qPCR MasterMix-LOW ROX™ following the manufacturer’s instructions. *β*-actin was used as an internal standard to normalize gene targets. Relative RNA expression was calculated using the 2^−∆∆CT^ method (Rieu and Powers 2009). The primers used were designed by Takara Biotechnology (Shiga, Japan) and are listed in Table 1. RT-qPCR experiments were carried out in triplicate.

**TABLE 1:** Primer sequences used in RT-qPCR.

Genes	Direction	Sequence (from 5′to 3′)
*β*-actin	Forward	TGG​CAC​CCA​GCA​CAA​TGA​A
	Reverse	CTA​AGT​CAT​AGT​CCG​CCT​AGA​AGC​A
Caspase-3	Forward	GAC​TCT​GGA​ATA​TCC​CTG​GAC​AAC​A
	Reverse	AGG​TTT​GCT​GCA​TCG​ACA​TCT​G
Bcl-2	Forward	AAC​ATC​GCC​CTG​TGG​ATG​AC
	Reverse	AGA​GTC​TTC​AGA​GAC​AGC​CAG​GAG
P53	Forward	TCG​AGA​TGT​TCC​GAG​AGC​TGA​AT
	Reverse	GTC​TGA​GTC​AGG​CCC​TTC​TGT​CTT
Bax	Forward	TTG​CTT​CAG​GGT​TTC​ATC​CA
	Reverse	CTT​GAG​ACA​CTC​GCT​CAG​CTT​C
Cytochrome C	Forward	GGA​GCG​AGT​TTG​GTT​GCA​CTT​AC
	Reverse	TGT​GGC​ACT​GGG​AAC​ACT​TCA​TA

### Statistical Analyses

Statistical analyses were undertaken using SPSS 19.0 (IBM, Armonk, NY, United States) applying a two-tailed independent Student’s *t*-test or one-way ANOVA followed by Dunnett’s *t*-test. Data are expressed as the mean ± standard error of the mean (SEM). *P* < 0.05 was considered statistically significant.

## Results

### Effects of Polysaccharides of Different Molecular Weights in a Zebrafish Xenograft Model

To determine the antitumor effects of *D*. *officinale* polysaccharides of different Mw on tumorigenesis *in vivo*, we established a novel zebrafish xenograft model by transplanting HT-29 cells. Zebrafish embryos (3 dpf) were treated with 5-Fu, D2, D8, DOPW-1, or D122.

**FIGURE 1:**
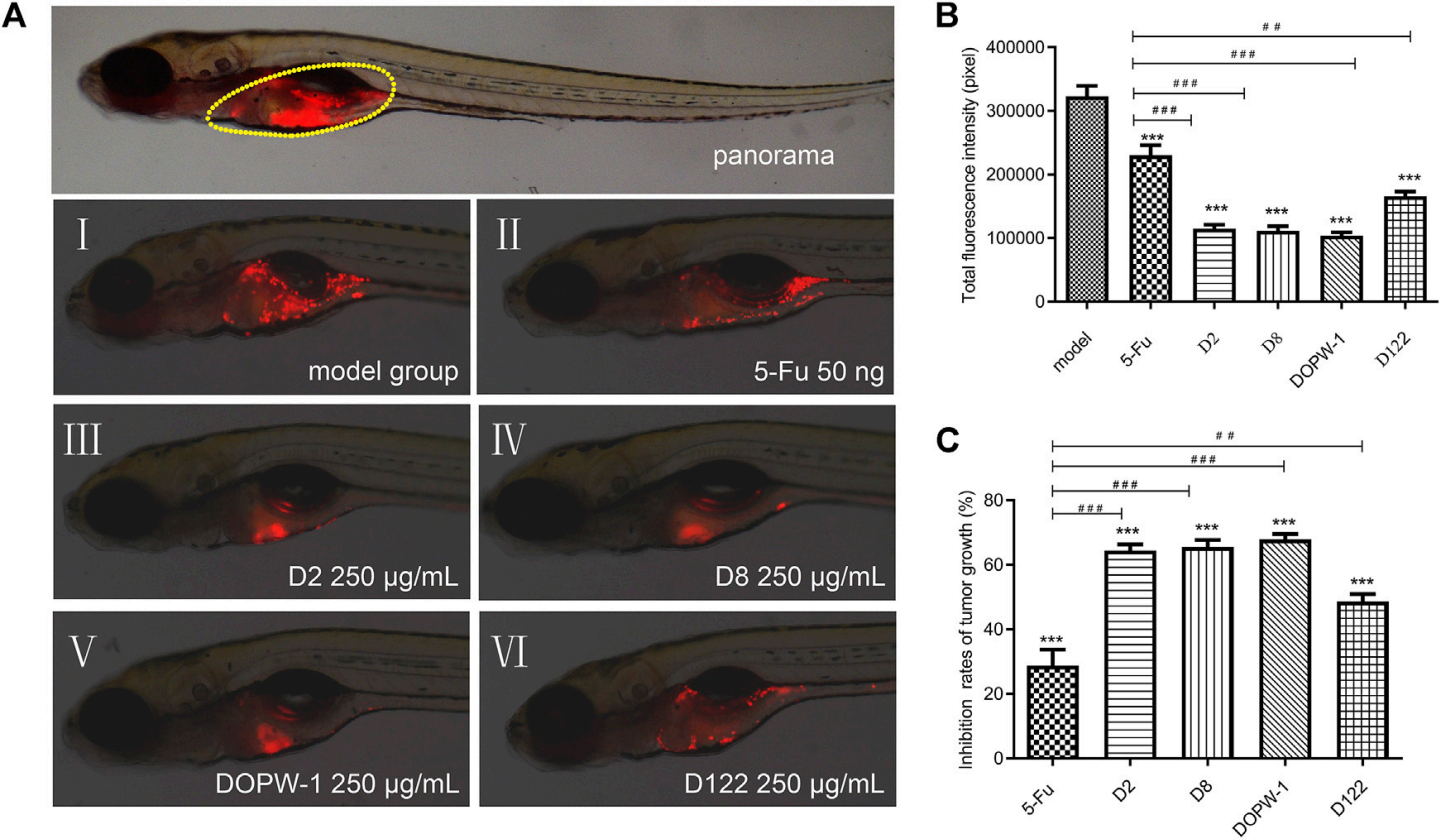
Inhibitory effect of polysaccharides of different Mw derived from *D*. *officinale* stems in a zebrafish xenograft model. **(A)** Red fluorescence represents a tumor area. **(B)** Fluorescence intensity of the tumor xenograft model. **(C)** Quantification of the inhibition of tumor growth. Data are expressed as the mean ± SEM. ^***^ *P* < 0.001 for each group *vs*. the model group; ^##^ *P* < 0.01 and ^###^ *P* < 0.001 for each group *vs*. the 5-Fu group.

The fluorescence intensity reflected the tumor volume. The inhibitory effect was revealed by reduced fluorescence intensity (Figure 1A). Notably, all treatments exhibited antitumor effects in the HT-29 xenograft model (Figures 1B,C). Compared with the model group, the inhibition of the tumor xenograft treated with 5-Fu, D2, D8, DOPW-1, or D122 was 28.77 ± 4.96%, 64.41 ± 1.90%, 65.49 ± 2.23%, 67.91 ± 1.69%, and 48.64 ± 2.23%, respectively (Table 2). Tumor growth was inhibited potently and significantly by 5-Fu, D2, D8, DOPW-1, and D122 (Figure 1). DOPW-1, with a Mw of 389.98 kDa, exhibited the strongest tumor inhibition.

**TABLE 2:** Effects of *D*. *officinale* polysaccharides of different Mw on a zebrafish xenograft model.

Group	Mean ± SEM	
	Fluorescence intensity (Pixels)	Inhibition (%)
Model	323075.80 ± 16458.07	0 ± 5.09
5-Fu (50 ng)	230121.44 ± 16011.40^a^	28.77 ± 4.96^a^
D2 (250 μg·ml^−1^)	114979.79 ± 6147.02^a^ ^,^ ^b^	64.41 ± 1.90^a^ ^,^ ^b^
D8 (250 μg·ml^−1^)	111484.58 ± 7199.76^a^ ^,^ ^b^	65.49 ± 2.23^a^ ^,^ ^b^
DOPW-1 (250 μg·ml^−1^)	103675.11 ± 5448.93^a^ ^,^ ^b^	67.91 ± 1.69^a^ ^,^ ^b^
D122 (250 μg·ml^−1^)	165928.09 ± 7214.14^a^ ^,^ ^c^	48.64 ± 2.23^a^ ^,^ ^c^

Data are expressed as the mean ± SEM.

a *P* < 0.001 for each group vs. the model group.

b *P* < 0.001 for each group vs. 5-Fu group.

c *P* < 0.01 for each group vs. 5-Fu group.

### Effects of *Astragalus membranaceus* Polysaccharides, *Dendrobium officinale* Kimura & Migo Polysaccharide-1, and *Lentinus edodes* Polysaccharides in a Zebrafish Xenograft Model

**FIGURE 2:**
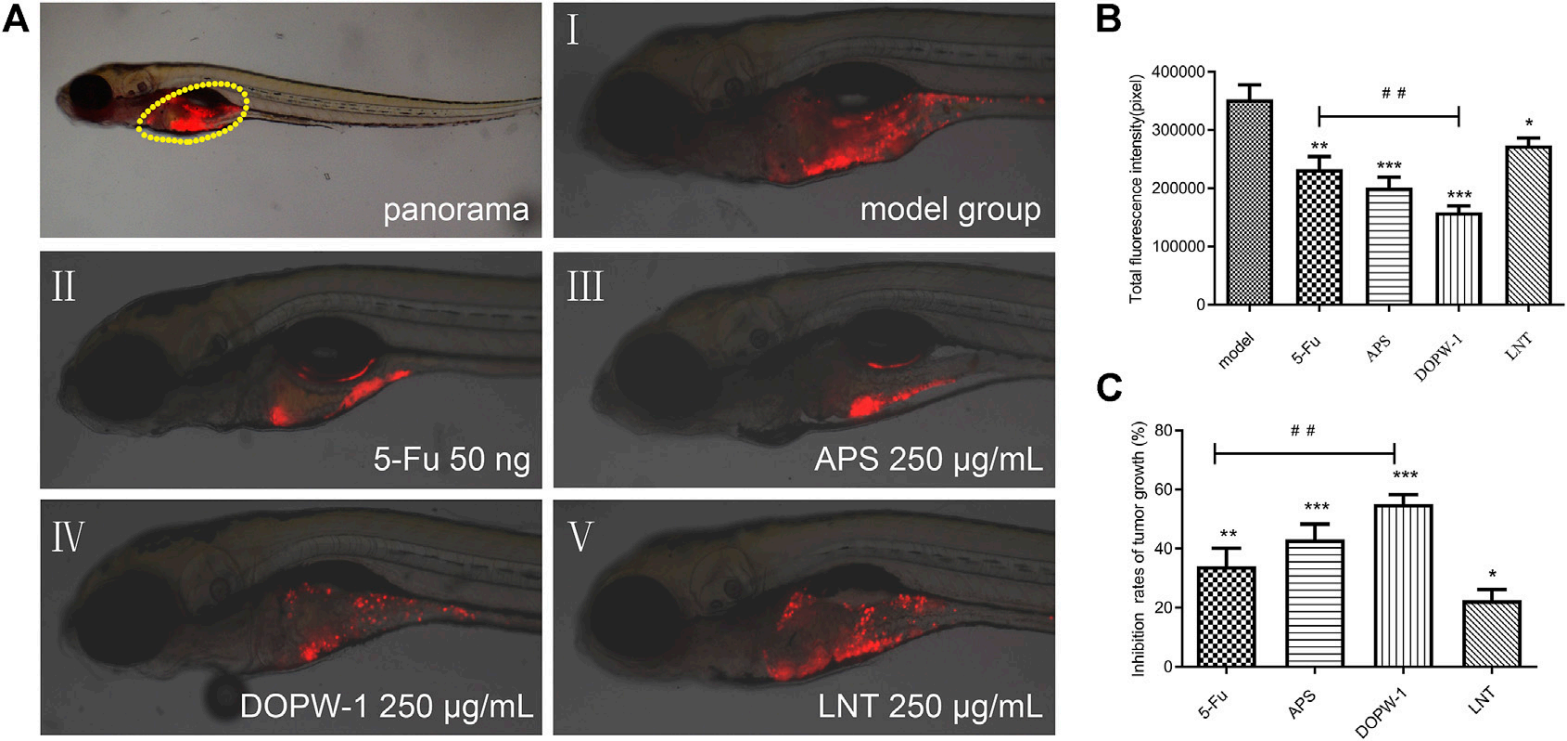
Tumor inhibition elicited by APS, DOPW-1, and LNT in a zebrafish xenograft model. **(A)** HT-29 cells (red) after xenotransplantation. **(B)** Fluorescence intensity of the tumor xenograft. **(C)** Quantification of the inhibition of tumor growth. Data are expressed as the mean ± SEM. ^*^ *P* < 0.05, ^**^ *P* < 0.01, and ^***^ *P* < 0.001 for each group *vs*. the model group; ^##^ *P* < 0.01 for the DOPW-1 group *vs*. the 5-Fu group.

The zebrafish model (created by implanting HT-29 cells) was also applied to determine the antitumor effects of APS, DOPW-1, and LNT on tumorigenesis *in vivo*. Zebrafish embryos (3 dpf) were treated with 5-Fu, APS, DOPW-1, or LNT. Growth inhibition was determined by capturing the fluorescence images using a fluorescence microscope. Figure 2A shows the tumor-inhibitory effect, which was indicated by reduced fluorescence intensity. Figures 2B,C show that all treatments exhibited antitumor effects in the zebrafish xenograft model. Compared with the model group, the inhibition of the tumor xenograft model treated with 5-Fu, APS, DOPW-1, or LNT was 34.03 ± 6.09%, 43.08 ± 5.19%, 55.13 ± 3.16%, and 22.48 ± 3.65%, respectively (Table 3). 5-Fu, APS, DOPW-1, and LNT inhibited tumor growth potently and significantly when compared with that in the model group (Figure 2). The inhibition of tumor growth elicited by DOPW-1 was significantly greater than that in the 5-Fu group. Moreover, compared with the APS and LNT groups, the DOPW-1 group exhibited the strongest tumor inhibition.

**TABLE 3:** Effects of APS, DOPW-1, and LNT on a zebrafish xenograft model.

Group	Mean ± SEM	
	Fluorescence intensity (Pixels)	Inhibition (%)
Model	352981.93 ± 24936.72	0 ± 3.89
5-Fu (50 ng)	232871.15 ± 21488.43^a^	34.03 ± 6.09^a^
APS (250 μg·ml^−1^)	200902.50 ± 18346.50^b^	43.08 ± 5.19^b^
DOPW-1 (250 μg·ml^−1^)	158395.32 ± 11137.22^b^ ^,c^	55.13 ± 3.16^b^ ^,c^
LNT (250 μg·ml^−1^)	273619.86 ± 12819.40^d^	22.48 ± 3.65^d^

Data are expressed as the mean ± SEM.

a *P* < 0.01.

b *P* < 0.001 for each group vs. the model group.

c *P* < 0.01 for the DOPW-1 group vs. the 5-Fu group.

d *P* < 0.05 for each group vs. the model group.

### Mapping of Reads

The strongest inhibitory effect on the growth of HT-29 cells was elicited by DOPW-1. Hence, DOPW-1 was selected as the experimental group to explore the difference in transcriptomes between it and the control group. All six constructed libraries (named control-1, control-2, control-3, DOPW-1-1, DOPW-1-2, and DOPW-1-3) were sequenced with raw reads.

An average of 52.39 (range, 46.14–63.26) million clean reads was obtained after discarding the adaptor sequence, poly-N–containing reads, and low-quality raw reads. HISAT2 was used to map clean reads to the human reference genome. A summary of the detailed mapping output is listed in Table 4. The ratio of mapping for the control and DOPW-1 groups was 94.69 and 94.71%, respectively, which demonstrated high gene expression in both groups.

**TABLE 4:** Summary of RNA-seq alignment.

Sample	Clean reads	Mapped reads	Uniquely mapped	Multiply mapped
Control-1	63,262,836	61,388,655 (97.04%)	59,818,229 (94.59%)	1,570,426 (2.48%)
Control-2	50,902,230	49,507,501 (97.26%)	48,243,322 (94.78%)	1,264,179 (2.48%)
Control-3	52,359,808	50,903,021 (97.22%)	49,597,105 (94.72%)	1,305,916 (2.49%)
DOPW-1-1	54,529,350	52,995,672 (97.19%)	51,623,823 (94.67%)	1,371,849 (2.52%)
DOPW-1-2	46,143,896	44,829,360 (97.15%)	43,666,343 (94.63%)	1,163,017 (2.52%)
DOPW-1-3	47,175,218	45,878,612 (97.25%)	44,744,159 (94.85%)	1,134,453 (2.40%)

Note: Control represents HT-29 cells without DOPW-1 treatment; DOPW-1 represents HT-29 cells with DOPW-1 treatment for 48 h.

### Functional Annotation and Differentially Expressed Genes Analysis

Analysis of gene function was performed using gene annotation. The genes were aligned against the publicly available protein databases KEGG, KOG, and GO. In total, 22,478 (96.88%) genes were annotated (Table 5). Most of the genes were annotated by gene functions using KOG (59.26%), KEGG (58.88%), and GO (51.75%) databases.

**TABLE 5:** Functional annotation of genes using protein databases.

Database	Number of genes annotated
GO	12,006 (51.75%)
KEGG	13,660 (58.88%)
KOG	13,749 (59.26%)
All annotations	22,478 (96.88%)
Total number of genes annotated	23201

**FIGURE 3:**
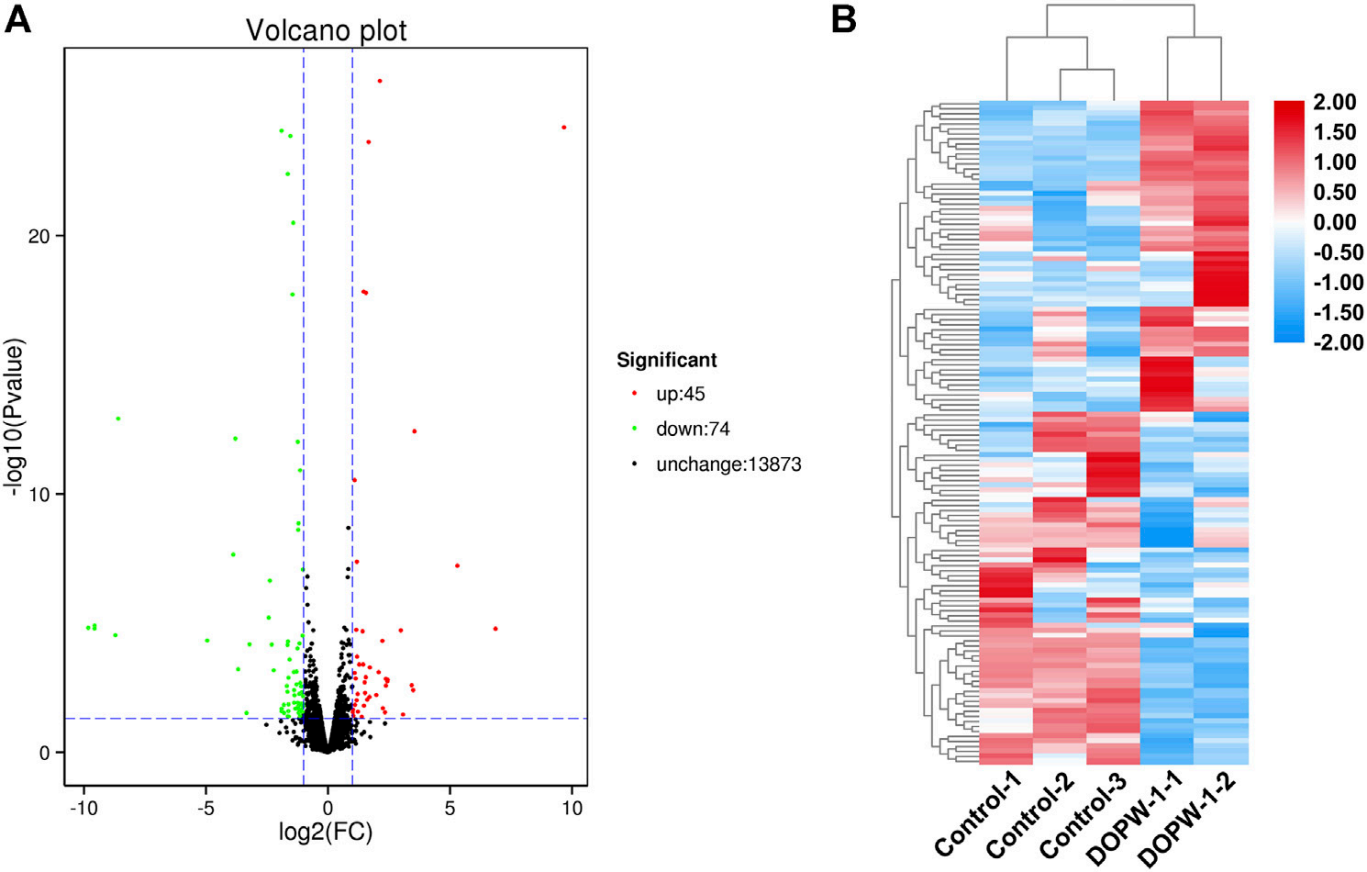
**(A)** Volcano map of DEGs between the DOPW-1 and control groups. Black, red, and green dots represent genes with no significant change in expression, significantly upregulated expression, and significantly downregulated expression in DEGs, respectively. **(B)** Hierarchical clustering of DEGs for the DOPW-1 group *vs*. the control group. Different colors represent different gene expressions in the DOPW-1 and control groups.

Hierarchical clustering was undertaken, and volcano plots were created to visualize the differential expression of genes (Figure 3A). We identified 119 genes with significant differential expression in the DOPW-1 group compared with that in the control group. Forty-five genes had upregulated expression and 74 genes had downregulated expression, which was likely due to activation of HT-29 cells after DOPW-1 treatment. Agglomerative hierarchical clustering was used to identify the number of clusters from the cluster dendrogram (Figure 3B).

### Functional Analysis of Genes Using Clusters of Protein Homology and Gene Ontology Databases

To evaluate and categorize the possible functions of genes, all unigenes were aligned to the KOG database. Orthologous genes were classified in the KOG database.

**FIGURE 4:**
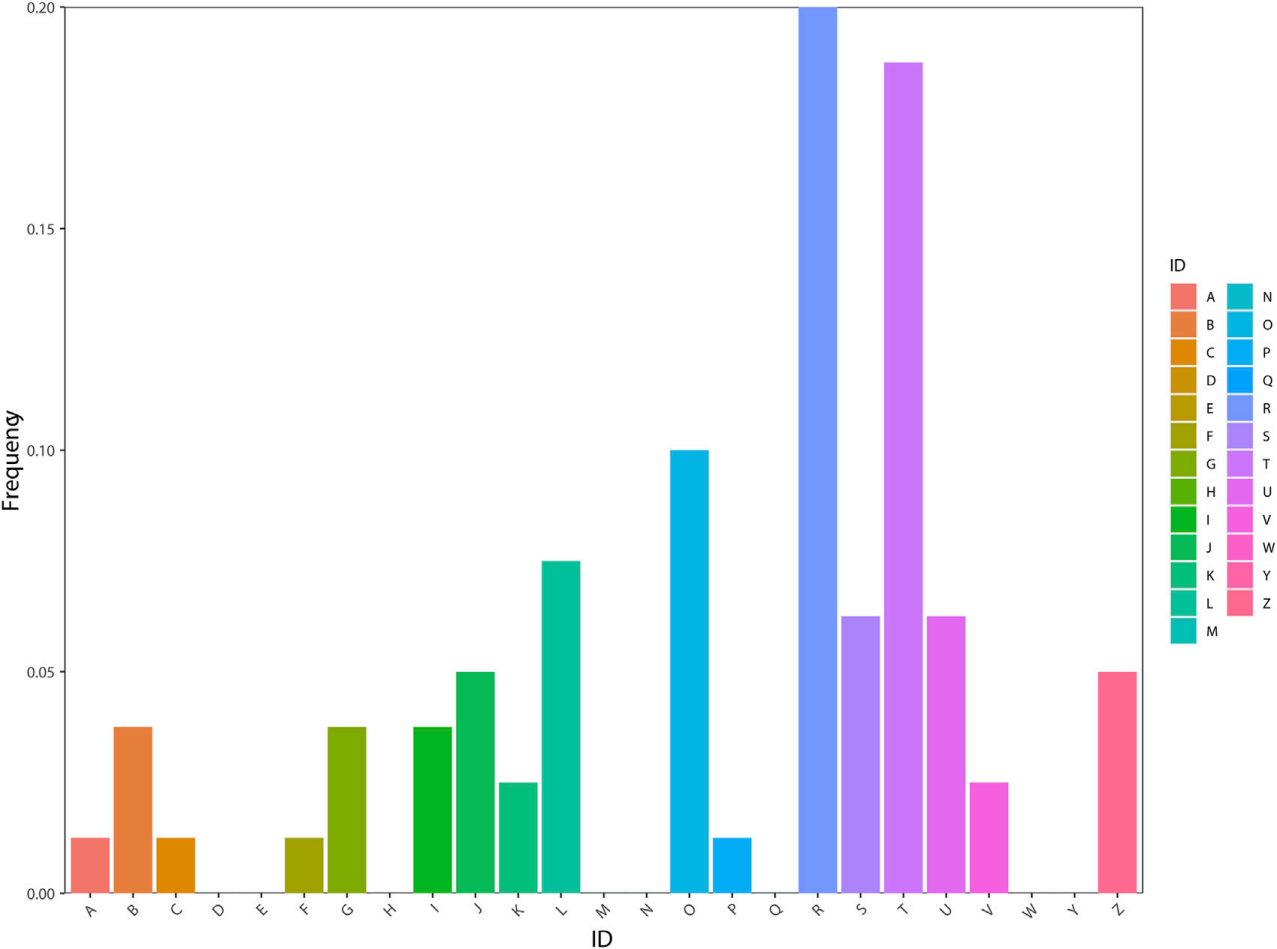
Functional classification of genes using the KOG database. **(A)** RNA processing and modification [1-1.25%]; **(B)** Chromatin structure and dynamics [3-3.75%]; **(C)** Energy production and conversion [1-1.25%]; **(D)** Cell cycle control, cell division, and chromosome partitioning [0-0%]; **(E)** Amino acid transport and metabolism [0-0%]; **(F)** Nucleotide transport and metabolism [1-1.25%]; **(G)** Carbohydrate transport and metabolism [3-3.75%]; **(H)** Coenzyme transport and metabolism [0-0%]; **(I)** Lipid transport and metabolism [3-3.75%]; **(J)** Translation, ribosomal structure, and biogenesis [4-5%]; **(K)** Transcription [2-2.5%]; **(L)** Replication, recombination, and repair [6-7.5%]; **(M)** Cell wall/membrane/envelope biogenesis [0-0%]; **(N)** Cell motility [0-0%]; **(O)** Posttranslational modification, protein turnover, and chaperones [8-10%]; **(P)** Inorganic ion transport and metabolism [1-1.25%]; **(Q)** Secondary metabolite biosynthesis, transport, and catabolism [0-0%]; **(R)** General function prediction only [16-20%]; **(S)** Function unknown [5-6.25%]; **(T)** Signal transduction mechanisms [15-18.75%]; **(U)** Intracellular trafficking, secretion, and vesicular transport [5-6.25%]; **(V)** Defense mechanisms [2-2.5%]; **(W)** Extracellular structures [0-0%]; **(Y)** Nuclear structure [0-0%]; **(Z)** Cytoskeleton [4-5%].

According to the KOG database, the genes were distributed in 17 orthologous clusters (Figure 4). Most of the genes were assigned to “general function prediction only” (16–20%), followed by “signal transduction mechanisms” (15–18.75%), “posttranslational modification, protein turnover, and chaperones” (8–10%), “replication, recombination, and repair” (6–7.5%), and “intracellular trafficking, secretion, and vesicular transport” (5–6.25%).

**FIGURE 5:**
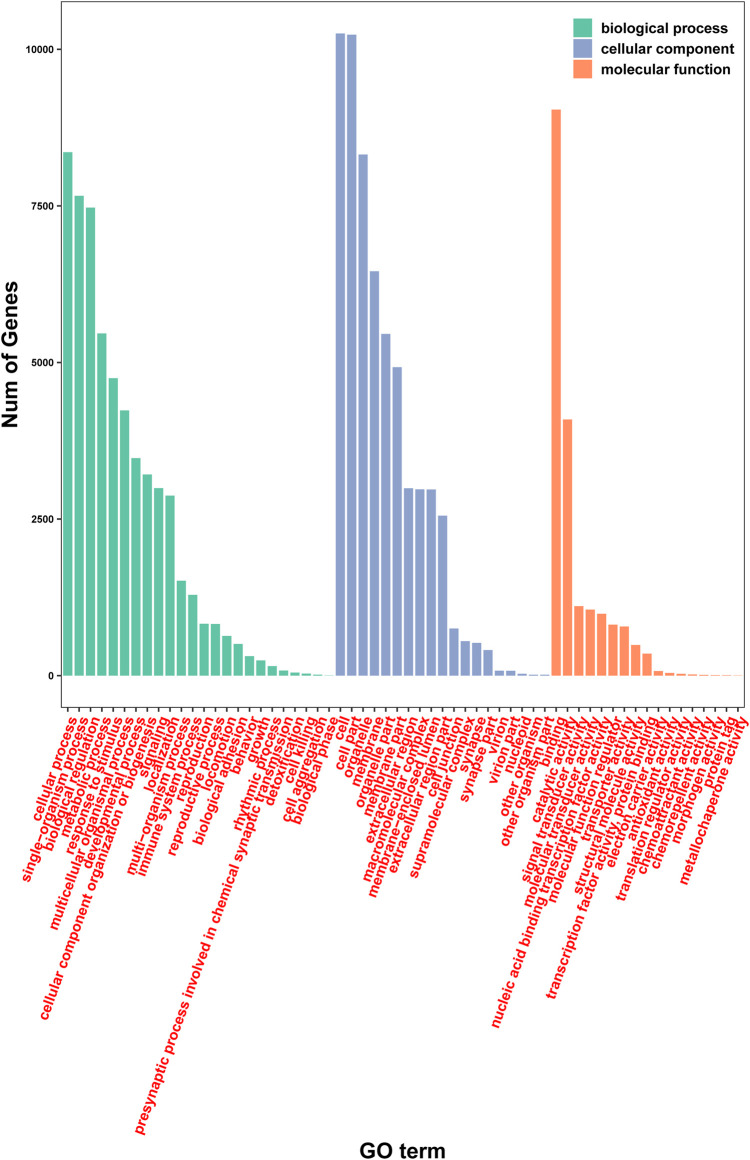
Analysis of gene function using the GO database.

Further analysis of gene function was performed using the GO database employing the categories of biological process, cell component, and molecular function. A total of 12,006 genes were mapped using the GO database (Figure 5). Specifically, 10,434, 11,381, and 10,623 genes were assigned to biological process, cell component, and molecular function, respectively. Importantly, several different GO terms may be assigned to the same gene.

### Analysis of Pathway Enrichment Using the Kyoto Encyclopedia of Genes and Genomes Database

**FIGURE 6:**
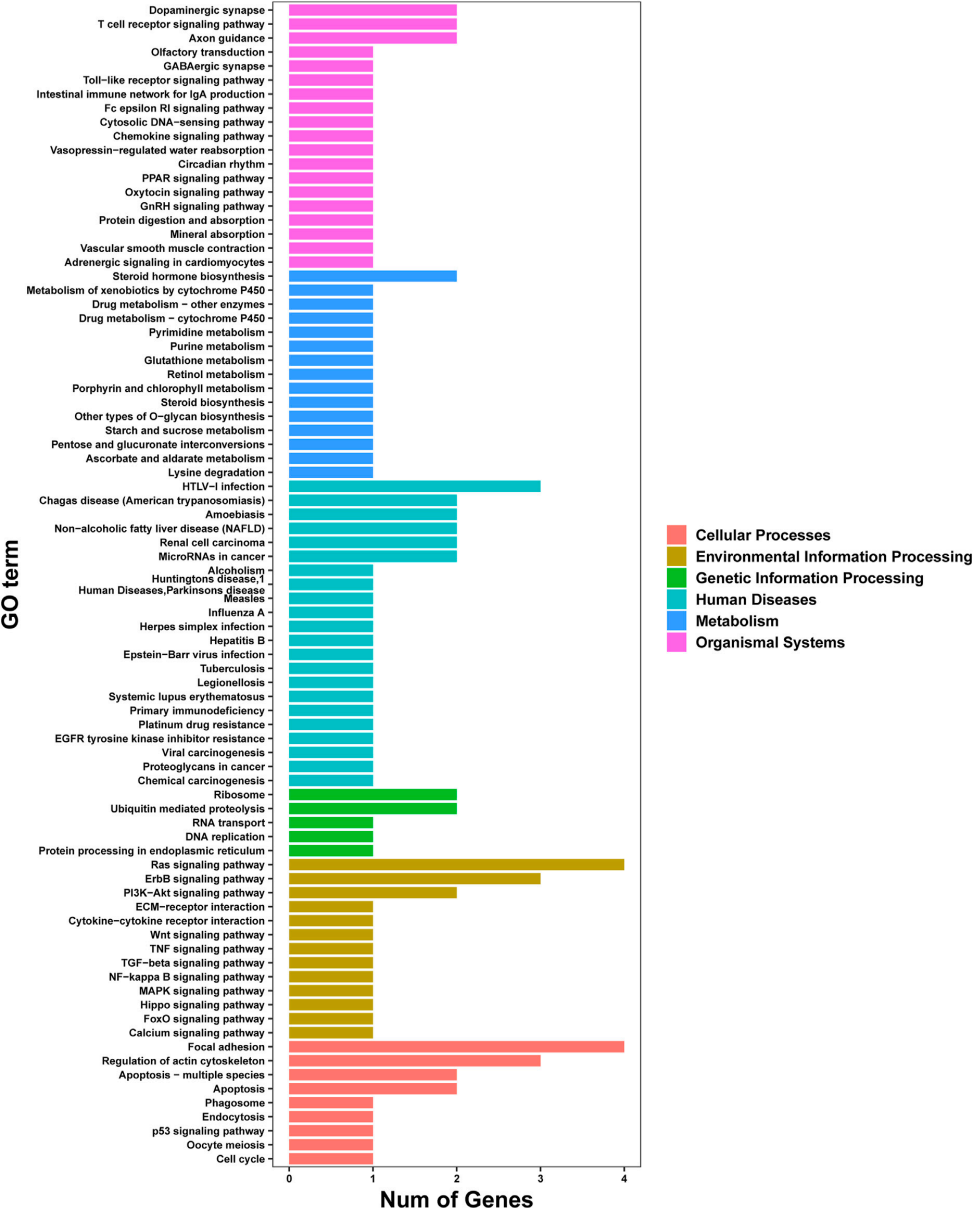
Analysis of pathway enrichment of genes using the KEGG database.

Analysis of pathway enrichment revealed most genes to be assigned to “apoptosis and apoptosis-multiple species” (12.5%), “focal adhesion” (12.5%), and “ras signaling pathway” (12.5%) followed by “regulation of actin cytoskeleton” (9.38%) and “ErbB signaling pathway” (9.38%) (Figure 6).

**FIGURE 7:**
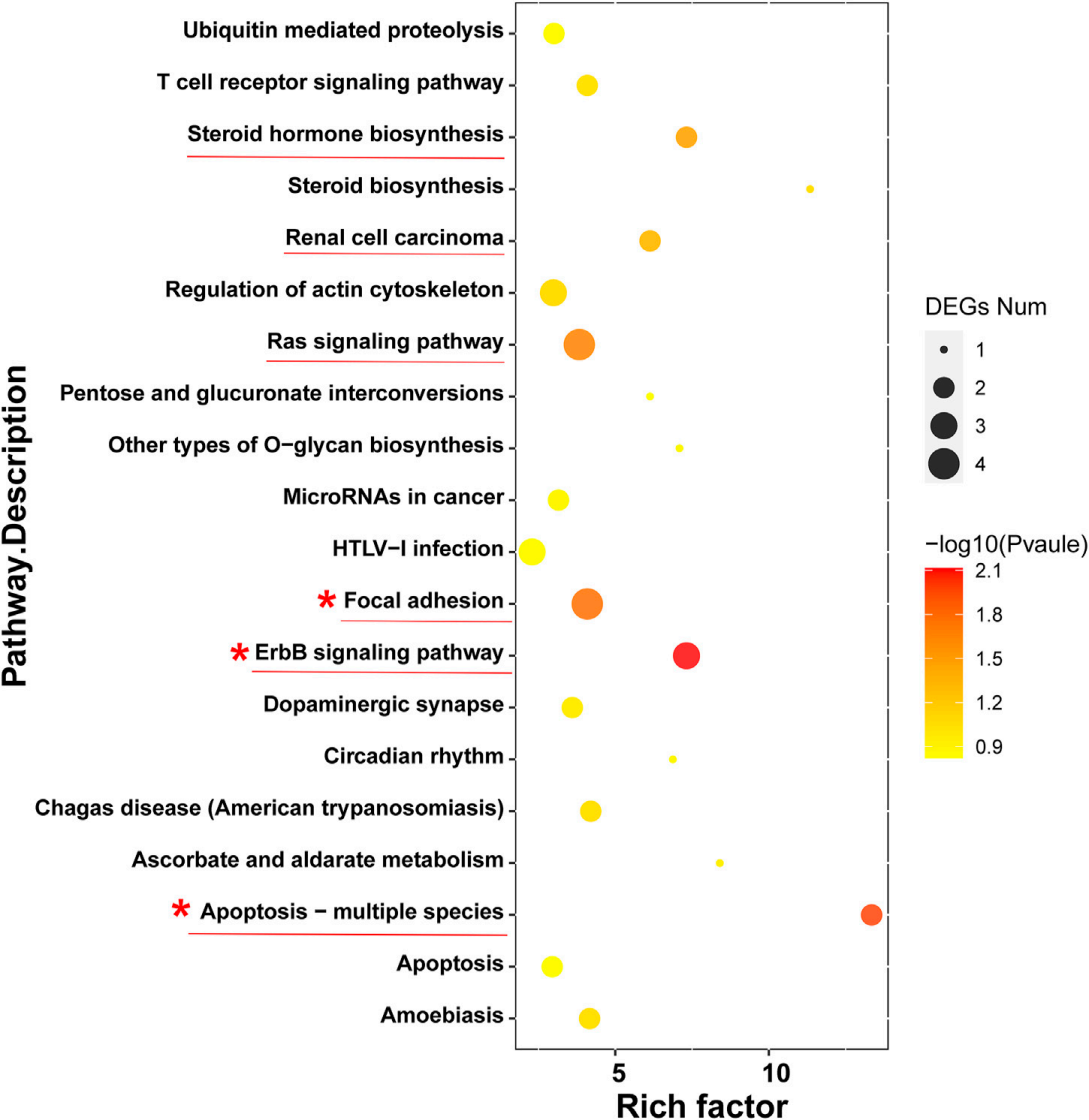
“Bubble diagram” of the top 20 ranked DEGs using the KEGG database.

To further investigate the anticancer signaling pathways of HT-29 cells treated with DOPW-1, we conducted enrichment analysis of DEGs using the KEGG database. The top 20 enriched pathways were selected (Figure 7). “Apoptosis-multiple species,” “ErbB signaling pathway,” “focal adhesion,” “ras signaling pathway,” “renal cell carcinoma,” and “steroid hormone biosynthesis” were the top six enriched pathways. Among them, “apoptosis-multiple species” was the most significantly enriched pathway. Hence, an apoptosis pathway is one of the most important pathways to consider if DOPW-1 inhibits the proliferation of HT-29 cells. This finding is consistent with an observation in our previous report (Tao et al., 2021).

### Transcriptome Analysis of “Apoptosis-Multiple Species”

**FIGURE 8:**
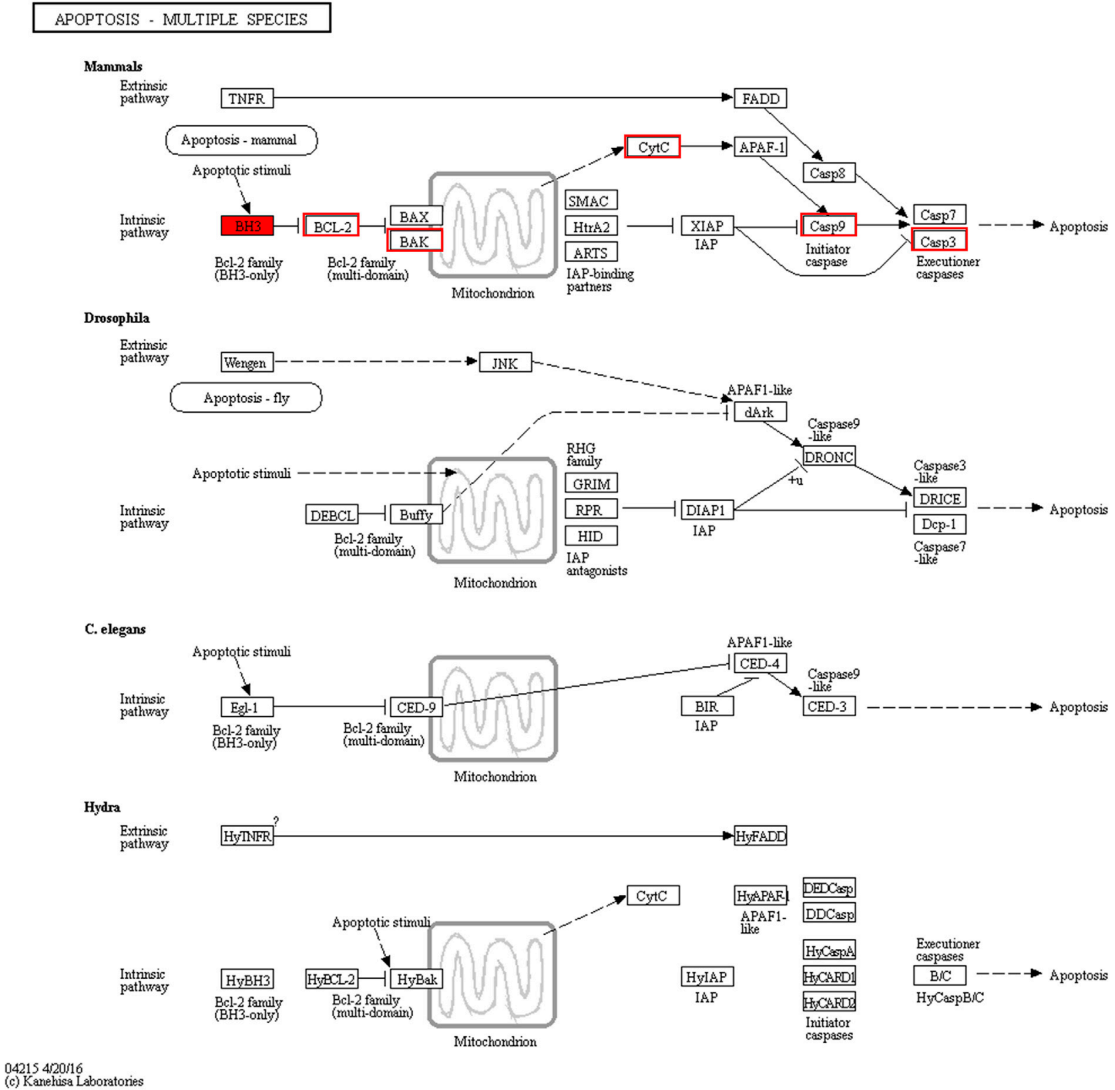
“Apoptosis-multiple species” pathway.

We discovered a close relationship among Bcl-2 family proteins, P53 protein, cytochrome C protein, caspase-3 protein, and apoptosis (Figure 8). In addition, the intrinsic pathway of apoptosis analyzed using RT-qPCR has been further verified by our previous study using Western blotting (Tao et al., 2021).

**FIGURE 9:**
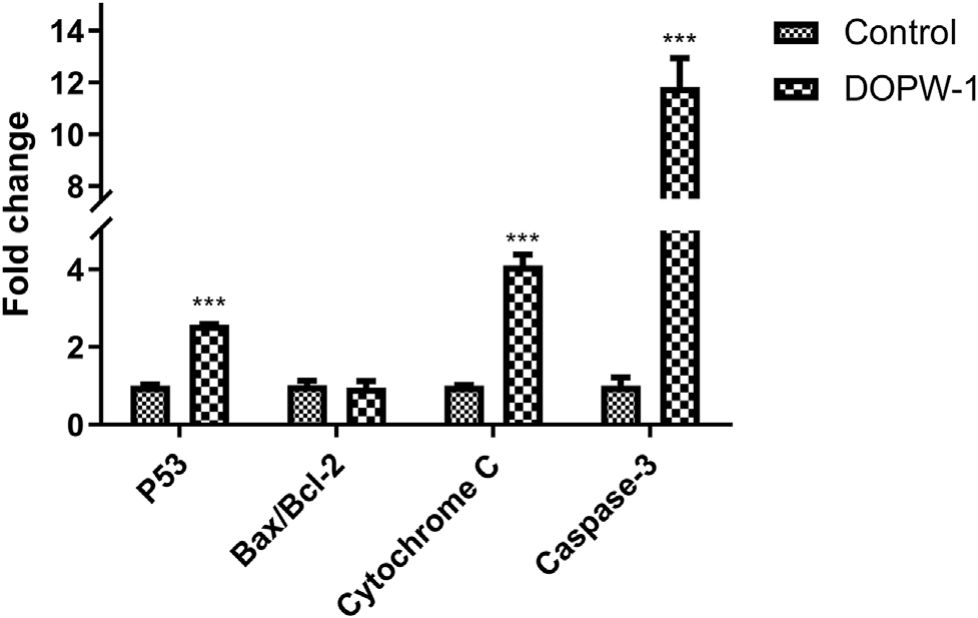
Effect of different treatments on gene expression. ^***^ *P* < 0.001 for the DOPW-1 group *vs*. the control group.

The apoptosis-related gene expression of P53, Bax, Bcl-2, cytochrome C, and caspase-3 was evaluated using RT-qPCR. Treatment with DOPW-1 (400 μg·ml^−1^) significantly increased the corresponding gene expression of P53, cytochrome C, and caspase-3 (Figure 9). Interestingly, the ratio of the expression of Bax/Bcl-2 in the DOPW-1 group was not significantly higher than that of the control group (*P* > 0.05). In our previous study, we calculated the ratio of the expression of Bak/Mcl-1 (anti- and pro-apoptotic factors, respectively) using Western blotting (Tao et al., 2021). Those results showed that the expression of Bak increased and that of Mcl-1 decreased after DOPW-1 treatment, which led to an increase in the Bak/Mcl-1 ratio when compared with that in the control group.

## Discussion

*D*. *officinale* polysaccharides possess anti-CRC activities (Tao et al., 2021). The Mw of polysaccharides is closely related to their antitumor activities (Huang et al., 2020). The side effects of chemotherapeutics warrant the development of nature-derived drugs based on polysaccharides. We investigated the effect of *D*. *officinale* polysaccharides of different Mw and currently used medicines based on polysaccharides (APS and LNT) in inhibiting tumor growth in a zebrafish xenograft model. We also studied the mechanism of action of *D*. *officinale* polysaccharides on the growth of HT-29 cells by RNA-seq.

In the study, the dose of *D*. *officinale* polysaccharides were selected based on our previous study (Tao et al., 2021), where we explored the MTC of polysaccharides from *D*. *officinale* and determined the effect of *D*. *officinale* polysaccharides (27.8, 83.3, 250 μg·ml^−1^) in a zebrafish xenograft model. The results indicated that the concentration of 250 μg·ml^−1^ is determined as the MTC and has the most significant inhibitory effect on CRC. *D*. *officinale* polysaccharides with a Mw ranging between 24.89 and 1,224.54 kDa exhibited significant antitumor activity, among which *D*. *officinale* polysaccharides of Mw 389.98 kDa exhibited the strongest tumor inhibition. This finding is in accordance with that of scholars who reported that lentinan of Mw 400–600 kDa exhibits strong antitumor activity (Wasser, 2002; Zhang et al., 2011). Also, Chen et al. (2009) postulated that polysaccharides of relatively high Mw may have more opportunity to “collide effectively”. Furthermore, long-chain polysaccharides have more repeating units and higher valency to bind to more receptors than that of short-chain polysaccharides. In addition, Zhang et al. (2016) reported that most polysaccharides of Mw from 10 kDa to 10^3^ kDa exhibited a marked antitumor effect. In the present study, D2 (24.89 kDa) and D8 (80.32 kDa) showed a strong antitumor effect, whereas D122 (1,224.54 kDa) showed a relatively weaker effect. One reason may be that polysaccharides of low Mw could enter tumor cells more readily than polysaccharides of high Mw. Yu et al. (2015) showed that high-Mw polysaccharides from *Porphyra yezoensis* were not conducive to inhibiting gastric cancer cells. Huang et al. (2020) revealed that high-Mw polysaccharides from *Dendrobium densiflorum* could not exert biological effects by breaking through membranes to enter cells. In general, the degradation of high-Mw polysaccharides to low-Mw polysaccharides can improve their biological activity significantly. Nevertheless, a too low Mw of polysaccharides will inhibit the formation of an active polymer structure.

Naturally derived polysaccharides have been widely used as medicines in China, thanks to their low toxicity and efficacy. For example, polysaccharides from *Astragalus* species, *Poria cocos*, *Ginseng* species, and *L*. *edodes* have been approved for clinical use by the National Medical Products Administration of China (Yu et al., 2018b). In the present study, the effect of DOPW-1 on CRC was more robust than that of the medicinal polysaccharides APS and LNT. Hence, DOPW-1 could be a promising potential agent against CRC. The effect of LNT on CRC was weaker than that of APS and DOPW-1, and the dose plays an important role in this result. Jeff et al. (2013) reported that the efficacious dose of LNT against the proliferation of HCT-116 cells and HT-29 cell was 5 mg·ml^−1^, whereas we employed an LNT dose of 250 μg·ml^−1^. Therefore, the anti-CRC activity of DOPW-1 was better than that of LNT at the same dose (250 μg·ml^−1^).

Transcriptome studies are used to explore the underlying molecular mechanisms of drug actions (Zhang et al., 2017). RNA-seq was undertaken to investigate how DOPW-1 inhibited the growth of HT-29 cells. Use of the KEGG database showed that the DEGs mainly encoded proteins involved in “apoptosis-multiple species,” “ErbB signaling pathway,” “focal adhesion,” “ras signaling pathway,” “renal cell carcinoma,” and “steroid hormone biosynthesis”. Among them, “apoptosis-multiple species” was the most significantly enriched. It is known that polysaccharides of *D*. *officinale* possess pharmacological activities of anticancerous nature (Yu et al., 2018a; Liang et al., 2019; Zhang et al., 2019), and this study might supply valuable information for DOPW-1 anti-CRC.

Apoptosis as an important molecular mechanism for polysaccharides in inhibiting colon cancer has been reported (Feregrino-Pérez et al., 2008; Di et al., 2017). For example, Di et al. (2017) reported that exopolysaccharides produced by *Lactobacillus casei* SB27 from yak milk induced the apoptosis of HT-29 cells. Feregrino-Pérez et al. (2008) revealed that a polysaccharide extract from common beans (*Phaseolus vulgaris* L.) could induce apoptosis via mitochondria-based mechanisms. In the present study, RT-qPCR demonstrated that gene expression of P53, cytochrome C, and caspase-3 was increased significantly after DOPW-1 treatment. However, the ratio of the expression of Bax/Bcl-2 in the DOPW-1 group was not significantly higher than that in the control group. Western blotting in our previous study showed that the expression of Bak increased and that of Mcl-1 decreased after DOPW-1 treatment, which led to an increase in the Bak/Mcl-1 ratio compared with that in the control group (Tao et al., 2021). Taken together, these results suggested that DOPW-1–triggered apoptosis of HT-29 cells was associated with the upregulation of the intrinsic p53 signaling pathway through caspase activation and, finally, led to apoptosis through the mitochondrial pathway (intrinsic pathway of apoptosis).

## Conclusion

Fractions of *D*. *officinale* polysaccharides with a Mw of 389.98 kDa may be the most suitable for antitumor effects against CRC. The antitumor effect of DOPW-1 on CRC was stronger than that of currently available alternatives: APS and LNT. RNA-seq revealed that an apoptosis pathway was important for DOPW-1 to inhibit the proliferation of HT-29 cells. We revealed that the pathway DOPW-1 induces the apoptosis of HT-29 cells and provided a scientific basis for use of DOPW-1 as a naturally occurring medicine against CRC. However, there are some limitations in this study. We merely performed the experiments in a zebrafish xenograft model, and clinical trials are needed to verify our data. On the other hand, a deeper understanding about the transcellular transport and subcellular location of *D*. *officinale* polysaccharides in CRC cells is yet to be gained. In the following studies, we will conduct further research to explore the transport and absorption of *D*. *officinale* polysaccharides in CRC cells by using labeling technology.

## Data Availability

The datasets presented in this study can be found in online repositories. The names of the repository/repositories and accession number(s) can be found below: https://www.ncbi.nlm.nih.gov/bioproject/PRJNA727088 PRJNA727088.

## Glossary

APS: *Astragalus membranaceus* polysaccharides
CRC: Colorectal cancer
DEGs: Differentially expressed genes
*D*. *officinale*: *Dendrobium officinale* Kimura & Migo
DOPW-1: *Dendrobium officinale* Kimura & Migo polysaccharide
FPKM: Fragments per kilobase of transcript per million mapped
GO: Gene Ontology
KOG: Clusters of protein homology
KEGG: Kyoto Encyclopedia of Genes and Genomes
LNT: *Lentinus edodes* polysaccharides
MTC: maximum tolerance concentration
Mw: molecular weight
RNA-seq: RNA sequencing
